# Older adults’ Internet use behavior and its association with accelerometer-derived physical activity

**DOI:** 10.3389/fpubh.2025.1537309

**Published:** 2025-02-12

**Authors:** Yen-Yu Chung, Jiaren Chen, Mei-Chun Lin, Ruo-Lan Liu, Jenn-Bang Wu, Hsin-Chang Tsai, Ting-Fu Lai, Yung Liao, Jong-Hwan Park

**Affiliations:** ^1^Graduate Institute of Sport, Leisure and Hospitality Management, National Taiwan Normal University, Taipei, Taiwan; ^2^Department of Physical Education and Sport Sciences, National Taiwan Normal University, Taipei, Taiwan; ^3^Department of Civic Education and Leadership, National Taiwan Normal University, Taipei, Taiwan; ^4^Holistic Education Center, National Taiwan Normal University, Taipei, Taiwan; ^5^Health Convergence Medicine Laboratory, Biomedical Research Institute, Pusan National University Hospital, Busan, Republic of Korea; ^6^Faculty of Sport Sciences, Waseda University, Tokorozawa, Japan; ^7^Department of Convergence Medicine, Pusan National University School of Medicine, Yangsan, Republic of Korea; ^8^Department of Clinical Bio-Convergence, Graduate School of Convergence in Biomedical Science, Pusan National University School of Medicine, Yangsan, Republic of Korea; ^9^Convergence Medical Institute of Technology, Pusan National University Hospital, Busan, Republic of Korea

**Keywords:** physical activity, Internet use, older adults, accelerometer, WHO

## Abstract

**Objective:**

The aging population is thriving worldwide, and it is critical to improve the health of older adults through physical activity (PA). Although the Internet can promote PA in older adults, limited studies have used objective tools to measure it. Thus, we aimed to investigate the association between the frequency of Internet use and PA levels in older adults.

**Methods:**

For this cross-sectional study, we employed convenience sampling. The participants were 172 adults aged 65 and older without cognitive impairment who could walk independently. We measured PA using a triaxial accelerometer, step counts, and moderate-to-vigorous PA (MVPA). We measured Internet use via a self-report questionnaire, separated by frequency of use into high, moderate, and low or no use. We performed a one-way analysis of variance (ANOVA) and multiple logistic regression to examine the relationship between Internet use and PA.

**Results:**

We included a total of 172 older adults (72.0 ± 5.5 years; 78.5% female). The group that used the Internet with moderate frequency exhibited higher daily step counts than the group that used the Internet with low frequency or not at all (moderate frequency: 7,888 steps > low frequency or no use: 6,070.6 steps). Compared to the group that used the Internet at a moderate frequency, older adults with a low frequency or those who did not use the Internet were less likely to meet the recommendations of the World Health Organization (WHO) regarding PA [odds ratio (OR): 0.242; 95% confidence interval (CI): 0.077–0.751].

**Conclusion:**

Older adults with a low frequency of Internet use or those who did not use the Internet (i.e., those who used the Internet less than once a week or not at all, respectively) were less likely to meet the WHO’s recommended levels for PA than older adults who used the Internet with moderate frequency. The findings of this study can inform efforts to reduce age-related health risks and promote strategies for encouraging PA.

## 1 Introduction

New developments in technology have changed the way we communicate. Easier access to computers and the increased use of smartphones have allowed people to utilize the Internet more frequently and conveniently. As of October 2023, there were 5.4 billion Internet users worldwide, comprising 67% of the global population (1). In the past decades, older adults were considered a vulnerable group regarding Internet use; they lacked the ability and opportunities to use the Internet, but as modernization spread around the globe, the situation changed (2, 3). Internet access and use among older adults are increasingly rising worldwide. Studies have demonstrated the benefits of and opportunities for Internet use among the older population, as the Internet offers great resources and services to promote older adults’ health, medications, daily needs, and social interactions (4). Because health-related issues are significant to the aging population, investigating Internet use and its relationship with healthy behaviors is imperative.

Engaging in physical activity (PA) can enhance the health of older adults’ health by decreasing their risk of cardiovascular diseases, type 2 diabetes, cancer, osteoporosis, sarcopenia, cognitive impairment, dementia, and depression (5). Based on these health benefits, the World Health Organization (WHO) suggests that older adults engage in 150–300 min of moderate physical activity (MPA), 75–150 min of vigorous physical activity (VPA) each week, or a combination of these (MVPA) (5). However, over 75% of community-dwelling older adults do not meet the WHO’s recommendations (6, 7). Therefore, encouraging older adults to increase their PA to achieve optimal health has become important in many countries.

Internet use provides benefits related to promoting PA and health status among older adults (8–10). The vast resources of the Internet have laid the foundation for guiding people toward a healthy lifestyle (11). Specifically, older adults can use the Internet to search for health-related information such as healthy behaviors, exercise instructions, and details of their diet and body composition (12, 13). Additionally, they can leverage social media or online communities to gain social support, which may motivate them to engage in healthy behaviors, or connect with peers and family members to participate in physical activities together. They can also use online resources to maintain social networks and manage PA (14–17). These mechanisms—enhancing health knowledge, self-efficacy, and social support—help promote physical activity among older adults.

Although numerous studies have discussed the correlation between Internet use and PA among adolescents and adults, most have collected PA data through self-report methods (18), which are susceptible to recall and social desirability bias. Moreover, only a few studies have focused on older adults. Hence, we focused on older adults and collected their PA data using a triaxial accelerometer. We aimed to determine whether there was a significant difference between the frequency of Internet use and PA. Furthermore, we aimed to investigate the association between the frequency of Internet use and the WHO’s recommended PA levels.

## 2 Materials and methods

### 2.1 Participants and study design

**Figure 1:**
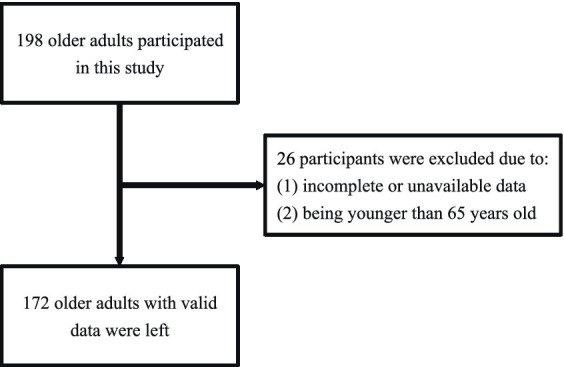
Flow of participants in the study.

Participants were recruited through convenience sampling using announcements from representatives in the neighborhood, senior citizen centers, and word-of-mouth from previous participants in Taipei, Taiwan, from May to August 2023. We included individuals who were (1) aged 65 years and older, (2) able to walk independently (we excluded those who needed mobility aids or those who received help from others), and (3) without cognitive impairment. All the participants provided informed consent and agreed to participate. Each participant was given a tri-axial accelerometer to wear for seven days and asked to record the duration when they did not wear it. We used approximately 30 accelerometers, which were cycled among participants throughout the study. They were informed about wearing the accelerometer properly and then responded to a structured questionnaire consisting of items on sociodemographic traits and Internet use. The questionnaire was interviewer-administered, with trained research personnel asking participants each question and assisting in recording their responses to ensure accuracy and understanding. A total of 198 older adults participated in this study; we excluded 26 with missing data and those aged <65 years, leaving 172 older adults with valid data (Figure 1). This study was approved by the Research Ethics Committee of National Taiwan Normal University (REC #202112HM024).

### 2.2 Objectively measured PA

We used a triaxial accelerometer (model wGT3X-BT; ActiGraph, Pensacola, FL, US) to measure PA and step count. The participants were advised to continuously wear the accelerometer on their waist for seven days; a valid day was defined as wearing the device for 10 or more hours while awake. Following previous studies, we included data from participants with four or more valid days (including one weekend day) in our analysis (19, 20). The device had to be removed when engaging in water-related activities, such as showering or swimming, and the participants were asked to record the times when the device was not worn. The data were processed following the standard methods described by Migueles, Cadenas-Sanchez (21), encompassing device placement, sampling frequency, valid days and weeks, epoch lengths, and definitions for non-wear periods. Specifically, considering the study participants were older adults and the accelerometer was worn on the waist, the sampling frequency was set at the standard 30 Hz for all data analyses. To include valid data, participants were required to have at least four valid days (≥600 min of wear time per day) (22). The epoch length was set at 60 s. Non-wear periods were defined as ≥60 consecutive minutes with zero counts, allowing for 1–2 min of interruptions with counts ranging from 0 to 100. Data from the accelerometers were processed and exported using ActiLife software (version 6.0, Pensacola, FL, US). PA comprised counts defined as equal to or higher than 100 counts per minute (CPM), which we classified as follows: (1) light PA (LPA), 100–2,019 CPM; (2) moderate PA (MPA): 2,020–5,998 CPM; and (3) vigorous PA (VPA): 5,999+ CPM (23). We also collected (4) step counts and included MPA, VPA, and daily step counts as exposure variables. To determine whether participants met the WHO recommendations, we combined the minutes of MPA and VPA into MVPA. We separated the participants into two groups: those who completed 150 min of MVPA per week and those who did not.

### 2.3 Frequency of internet use

Internet use in this study was defined as the frequency of accessing online resources or engaging in activities through the Internet, regardless of the purpose. The questionnaire is a simplified version of the Digital Development Survey administered by Taiwan’s Ministry of Digital Affairs in 2022 (24). Respondents disclosed their Internet usage by selecting from predefined options: 0 = no knowledge or refusal to respond, 1 = less than once a week, 2 = 1–3 days, 3 = 4–6 days, 4 = daily use of the Internet for short periods (low frequency), and 5 = daily use of the Internet for extended periods (high frequency). In line with the Digital Development Survey (24), we aggregated the responses into three categories: 0 and 1 = low frequency or no use; 2–4 = moderate; and 5 = high.

### 2.4 Covariates

We collected demographic information was collected using self-report questionnaires. The covariates included age, sex, marital status (married or unmarried), living arrangements (living alone or with others), educational level (tertiary education consisting of a university/college degree or higher), and employment status (full-time vs. non-full-time).

### 2.5 Statistical analyses

We performed a descriptive analysis to shed light on the participants’ characteristics, highlighting sociodemographic variables such as age, sex, marital status, living arrangements, education level, and employment status. Additionally, chi-square tests were conducted to explore the relationships between sex, adherence to the WHO’s recommendation of 150 min of MVPA per week, and the frequency of Internet use. We detailed the proportion of participants who did and did not meet the WHO’s recommended 150 min of weekly MVPA. We expressed the data for the frequency of Internet use (low or no use, moderate, and high) and PA (step count, LPA, MPA, and VPA) through means and standard deviations (SDs). We examined the link between the frequency of Internet use and PA levels using a one-way analysis of variance (ANOVA) to determine the statistical significance of differences between the groups. Furthermore, we performed multiple logistic regression to explore the association between the frequency of Internet use and the completion of at least 150 min of MVPA each week. We conducted all statistical analyses using IBM SPSS software, version 26.0 (SPSS Inc., IBM, Chicago, IL, US), with a significance threshold at *p* < 0.05.

## 3 Results

### 3.1 Participants characteristics

We included a total of 172 older adults in the study. Their mean age was 72 years (SD = 5.5). Most participants were aged 65–74 (73.3%), 78.5% were female, 82.0% were living with others, 85.5% were married, 59.9% had a university/college degree or higher, 93.6% were not in full-time employment, and 87.8% possessed internet usage skills. Chi-square tests revealed no statistically significant associations between sex and adherence to the WHO’s recommendation of 150 min of MVPA per week (*p* = 0.823) or sex and the frequency of Internet use (*p* = 0.843). They walked an average of 7214.41 (SD = 3104.24) steps per day and engaged in 0.26 (SD = 1.29) minutes of VPA per day, 21.15 (SD = 18.649) minutes of MPA per day, and 289.51 (SD = 73.60) minutes of LPA per day (Table 1). Table 2 presents the results of the comparison of PA in terms of physical activity among the three groups. We found a significant difference in the step count (*F* = 3.320; *p* < 0.039). The *post hoc* test showed that the step count values were significantly lower in the group with low frequency of use or no use than in the group with a moderate frequency of use.

**Table 1:** Characteristics of the participants (*n* = 172).

Sociodemographic variables	*n*	%
Age		
65–74	126	73.3
≥75	46	26.7
Sex		
Female	135	78.5
Male	37	21.5
Marital status		
Married	147	85.5
Unmarried	25	14.5
Living arrangements		
With others	141	82
Living alone	31	18
Education level		
Tertiary education	103	59.9
No tertiary education	69	40.1
Employment status		
Full-time	11	6.4
Non-full-time	161	93.6
Meeting the WHO’s recommended 150 min of weekly MVPA		
Yes	67	39
No	105	61

Continuous variables	Mean	SD
Step count (steps/day)	7214.41	3104.24
VPA (minutes/day)	0.26	1.29
MPA (minutes/day)	21.15	18.65
LPA (minutes/day)	289.52	73.60

LPA, light physical activity; MPA, moderate physical activity; SD, standard deviation; VPA, vigorous physical activity; WHO, World Health Organization.

**Table 2:** Comparison of PA in older adults for the three groups using one-way analysis of variance (ANOVA) followed by *post-hoc* analysis.

Variables	Frequency of Internet use			*F*	*p*	*Post-hoc*
	Low frequency or no use ^a^ (*n* = 28)	Moderate ^b^ (*n* = 57)	High ^c^ (*n* = 87)			
Step count	6070.59 (3103.25)	7880.01 (3294.37)	7146.46 (2888.92)	3.32	0.039*	b > a
VPA	0.07 (0.32)	0.41 (2.06)	0.22 (0.71)	0.75	0.47	
MPA	14.98 (18.54)	23.24 (18.48)	21.78 (18.58)	1.96	0.14	
LPA	276.14 (66.88)	294.23 (80.64)	290.73 (71.11)	0.59	0.56	

Post-hoc: Scheffe, Bonferroni, Tukey’s honestly significant difference (HSD) and Fisher’s least significant difference (LSD) tests were used to correct for multiple comparisons. The data are represented by the mean (SD); *p < 0.05. LPA, light physical activity; MPA, moderate physical activity; VPA, vigorous physical activity.

### 3.2 The association between the frequency of Internet use and PA

Table 3 showed the multiple logistic regression analysis, using the group with a moderate frequency of use as a reference, we observed significant associations for the group with a low frequency of use or no use across all models. In Model I, older adults in the group with a low frequency of use or no use exhibited a 70% decrease in the odds of achieving the WHO-recommended 150 min of weekly MVPA compared to the group with a moderate frequency of use (odds ratio [OR]: 0.303; 95% confidence interval [CI]: 0.107–0.859). Model II, adjusted for age and sex, showed a 66% reduction in the odds for the group with a low frequency of use or no use relative to the group with a moderate frequency of use (OR: 0.340; 95% CI: 0.118–0.977). Finally, Model III, adjusted for the full set of covariates (age, sex, marital status, living arrangements, education level, and employment status), demonstrated that the odds were 76% lower for the group with low frequency of use or no use to meet the WHO’s recommended 150 min of weekly MVPA compared to the group with moderate frequency of use (OR, 0.242; 95% CI, 0.077–0.751).

**Table 3:** The association between the frequency of Internet use and the WHO’s recommended MVPA levels in older adults.

Frequency of Internet use	The WHO’s recommended MVPA levels in older adults ( ≧ 150 min / per week)								
	Model I			Model II			Model III		
	OR	95% CI	*p*	OR	95% CI	*p*	OR	95% CI	*p*
Low or no use	0.303	(0.107, 0.859)	0.025*	0.34	(0.118, 0.977)	0.045*	0.241	(0.077, 0.751)	0.014*
Moderate	Ref	-	-	Ref	-	-	Ref	-	-
High	0.713	(0.363, 1.400)	0.326	0.67	(0.337, 1.329)	0.251	0.618	(0.302, 1.265)	0.188

OR, odds ratio; CI, confidence interval.

Model II: adjusted for age, sex.

Model III: adjusted for age, sex, living arrangements, marital status, education level, employment status.

Ref, reference group; serves as the baseline category for comparison in the logistic regression analysis.

*p < 0.05.

## 4 Discussion

In this study, we analyzed the relationship between the frequency of Internet use and objectively measured PA among older adults. Although some previous studies have investigated the association between Internet use among older adults and PA (25–29), this study is one of the first to use an objectively measured tool to collect PA data and examine its association with Internet use among older adults. Our primary finding was that Internet use in older adults was associated with health-enhancing PA levels. This outcome not only provides supportive evidence for previous research but also valuable insights for health promotion practitioners and policymakers aiming to enhance PA in the older population (30).

Based on the demographic characteristics of our sample, the majority were older females, married, living with others, and not engaged in full-time employment, reflecting a sociodemographic profile similar to that reported in previous studies conducted in Taiwan (31, 32). Notably, 61% of participants did not meet the WHO’s recommended 150 min of weekly MVPA, underscoring the importance of understanding the relationship between Internet use and PA among older adults in Taiwan to effectively promote physical activity. Additionally, 87.8% of participants in this study possessed internet usage skills. This proportion is higher than the average internet usage rate for older adults in Taiwan (33), likely because participants were recruited in Taipei City, where internet penetration and digital literacy rates are generally higher.

Our main finding of this study is that after adjusting for potential confounders, compared with the group with a moderate frequency of use, older adults with low frequency of use or no use were less likely to meet the WHO’s recommendation of 150 min of weekly MVPA (OR = 0.241, 95% CI = 0.077–0.751, *p* = 0.014; Model III; Table 3). This is consistent with several prior studies that have used self-reported PA questionnaires to examine this issue (12, 34, 35). However, our results differed between adolescents and younger adults. Frequent Internet use may lead to negative impacts and reduced time spent on PA among younger populations (36–39). One possible explanation is that the younger and older populations have different reasons for using the Internet. Younger people lean toward activities such as social media engagement, instant messaging, and sharing multimedia content (mostly videos) (39–41). By contrast, older adults tend to search for health-related information (including PA) that supports their engagement in health-boosting behaviors (12, 42, 43). From the perspective of the health communication theory, older adults with the ability to use the Internet can actively gather health-related information. This may increase their knowledge and willingness to participate in activities that help increase their PA (44, 45). Therefore, longitudinal studies should be conducted to explore the impact of both Internet use and PA on older adults.

In addition, we noted a significant difference in step count across various frequencies of Internet use (Table 2). This finding is similar to those of previous studies (46, 47). Compared to older adults with a low frequency of use or no use, the group with a high frequency of use showed no significant difference, whereas the group with a moderate frequency of use (once a week to almost daily) exhibited significantly higher step counts (*F* = 3.32, *p* = 0.039; Table 2). One possible reason for this is that older adults with the ability to use the Internet may have greater access to information about the benefits of PA; therefore, they develop a deeper awareness of its importance (48). Social relationships are associated with higher daily step counts among older adults (49). Moreover, the Internet enables older adults to build strong social networks and increase social interactions by connecting with family and friends (50), helping them maintain a sense of belonging and receive social support (51). Despite these benefits, excessive Internet use may be unhealthy (52). Hamer and Stamatakis (53) found that using the Internet frequently decreased the number of outside activities performed by older adults. Consequently, Wang et al. (29) indicated that moderate Internet use is beneficial for older adults. Our research also supports this finding: a moderate frequency of use could promote PA among older adults. Thus, a moderate frequency of use is beneficial for older adults, not only for enhancing their social interactions but also for increasing their PA levels.

The strengths of this study include being the first to explore the association between the frequency of Internet use and objectively measured PA levels using a triaxial accelerometer, thereby providing reliable evidence for the relationship between Internet use and PA in older adults. However, this study had some limitations. First, because this study adopted a cross-sectional design, it is not possible to establish a causal relationship between the examined variables. Second, our participants were recruited through convenience sampling, which may have introduced a sampling bias owing to the regional source of the sample. Participants may share similar characteristics such as marital status, education level, employment status, and frequency of Internet use; therefore, the findings cannot be generalized to the broader older population in Taiwan. Additionally, the disproportionate sex distribution in our sample, with a higher proportion of older females than males, may be partially explained by older females in the community being somewhat more inclined to participate in research studies compared to males. Although chi-square tests showed no statistically significant association between sex and the key variables examined, this imbalance may still limit the generalizability of our findings. Future studies should aim for a more balanced sex distribution to improve the robustness and applicability of the results. Third, the frequency of Internet use was self-reported, which may lead to discrepancies between subjective perceptions and objective usage patterns. Future studies should consider incorporating objective measurements, such as health apps or non-invasive ecological momentary assessments (EMA) via sensors embedded in smartphones, alongside self-report questionnaires. This combined approach could offer a more comprehensive understanding of Internet use frequency and its relationship with PA. Additionally, future studies should expand the investigation to include the specific purposes or types of Internet use among older adults. Such an approach would enable a deeper exploration of the mechanisms linking Internet use to PA, providing insights into whether different purposes of Internet use (e.g., health information seeking, social networking) are associated with variations in PA levels. Understanding these specific mechanisms could inform tailored interventions to promote PA among older adults.

## 5 Conclusion

Compared to the group with a moderate frequency of use (once a week to almost daily), older adults with a low frequency of use or no use were less likely to meet the WHO’s recommended PA levels. Hence, offering courses on the Internet to older adults with low or no frequency of use may be helpful in promoting PA. Additionally, moderate use should be encouraged, as it is effective in increasing PA. Furthermore, this study may serve as a reference for future health-promotion programs for older adults.

## Data Availability

The original contributions presented in the study are included in the article/supplementary material, further inquiries can be directed to the corresponding author.
